# Prediction of Clinical Precision Chemotherapy by Patient‐Derived 3D Bioprinting Models of Colorectal Cancer and Its Liver Metastases

**DOI:** 10.1002/advs.202304460

**Published:** 2023-11-16

**Authors:** Hang Sun, Lejia Sun, Xindi Ke, Lijuan Liu, Changcan Li, Bao Jin, Peipei Wang, Zhuoran Jiang, Hong Zhao, Zhiying Yang, Yongliang Sun, Jianmei Liu, Yan Wang, Minghao Sun, Mingchang Pang, Yinhan Wang, Bin Wu, Haitao Zhao, Xinting Sang, Baocai Xing, Huayu Yang, Pengyu Huang, Yilei Mao

**Affiliations:** ^1^ Department of Liver Surgery Peking Union Medical College (PUMC) Hospital Peking Union Medical College (PUMC) & Chinese Academy of Medical Sciences (CAMS) Beijing 100730 China; ^2^ Department of General Surgery The First Affiliated Hospital Nanjing Medical University Nanjing Jiangsu 210029 China; ^3^ The First School of Clinical Medicine Nanjing Medical University Nanjing Jiangsu 210029 China; ^4^ Department of Hepatopancreatobiliary Surgery I Key Laboratory of Carcinogenesis and Translational Research (Ministry of Education) Peking University Cancer Hospital & Institute Beijing 100142 China; ^5^ Department of General Surgery The First Affiliated Hospital of USTC Division of Life Sciences and Medicine University of Science and Technology of China Hefei Anhui 230001 China; ^6^ Department of Hepatobiliary Surgery National Cancer Center/National Clinical Research Center for Cancer/Cancer Hospital Chinese Academy of Medical Sciences and Peking Union Medical College Beijing 100021 China; ^7^ First Department of Hepatopancreatobiliary Surgery China‐Japan Friendship Hospital Beijing 100029 China; ^8^ Chinese Academy of Medical Sciences and Peking Union Medical College Beijing 100730 China; ^9^ Department of General Surgery Peking Union Medical College (PUMC) Hospital Peking Union Medical College (PUMC) & Chinese Academy of Medical Sciences (CAMS) Beijing 100730 China; ^10^ State Key Laboratory of Advanced Medical Materials and Devices Engineering Research Center of Pulmonary and Critical Care Medicine Technology and Device (Ministry of Education) Institute of Biomedical Engineering Chinese Academy of Medical Science & Peking Union Medical College Tianjin 300192 China; ^11^ Tianjin Institutes of Health Science Tianjin 301600 China

**Keywords:** 3D bioprinting, cancer model, colorectal cancer, colorectal cancer liver metastases, individualized therapy, precision medicine

## Abstract

Methods accurately predicting the responses of colorectal cancer (CRC) and colorectal cancer liver metastasis (CRLM) to personalized chemotherapy remain limited due to tumor heterogeneity. This study introduces an innovative patient‐derived CRC and CRLM tumor model for preclinical investigation, utilizing 3d‐bioprinting (3DP) technology. Efficient construction of homogeneous in vitro 3D models of CRC/CRLM is achieved through the application of patient‐derived primary tumor cells and 3D bioprinting with bioink. Genomic and histological analyses affirm that the CRC/CRLM 3DP tumor models effectively retain parental tumor biomarkers and mutation profiles. In vitro tests evaluating chemotherapeutic drug sensitivities reveal substantial tumor heterogeneity in chemotherapy responses within the 3DP CRC/CRLM models. Furthermore, a robust correlation is evident between the drug response in the CRLM 3DP model and the clinical outcomes of neoadjuvant chemotherapy. These findings imply a significant potential for the application of patient‐derived 3DP cancer models in precision chemotherapy prediction and preclinical research for CRC/CRLM.

## Graphical abstract

The study showcases the establishment of patient‐derived 3D bioprinting models for colorectal cancer and its liver metastases. These models highly retain parent tumor biomarkers and mutation profiles, revealing substantial heterogeneity. Crucially, drug testing correlates strongly with clinical response, highlighting the great potential of 3D bioprinting tumor model as a preclinical platform for personalized cancer therapy.

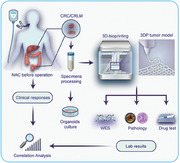

## 1 Introduction

Colorectal cancer (CRC) is a lethal cancer with a high fatality rate worldwide.^[^ ^1^ ^]^ Approximately 2 million people suffer from this cancer, with almost 900 000 deaths related to CRC occurring annually, with a growing trend in some countries.^[^ ^2^, ^3^ ^]^ Due to limitations in early diagnosis, a significant number of patients are in the advanced stage when diagnosed. Almost 20% of these patients have liver metastases (CRLM). Liver metastasis is the most common form of metastasis in CRC and is the leading cause of death in patients with CRC.^[^ ^1^, ^2^ ^]^

With early detection through screening and with continuous improvement in treatment methods, including surgery, radiotherapy, chemotherapy, and targeted therapy, the overall survival rate of CRC patients has improved greatly in recent years, as compared with the past.^[^ ^2^, ^4^, ^5^ ^]^ Systemic chemotherapy with 5‐fluorouracil (5‐FU) combined with oxaliplatin or irinotecan (CPT‐11) has always played a key role in adjuvant therapy for CRC, improving survival in patients with resected CRC.^[^ ^6^, ^7^ ^]^ For CRLM patients, surgical resection is considered to be the preferred treatment modality,^[^ ^8^, ^9^ ^]^ which may provide better survival benefits for patients when combined with systemic chemotherapy.^[^ ^10^, ^11^, ^12^, ^13^ ^]^

Nevertheless, CRC has high levels of heterogeneity,^[^ ^14^ ^]^ and chemotherapy efficacy remains limited. Therefore, individualized treatment plans, particularly for patients with metastatic CRC, have become increasingly important and urgent. Unfortunately, given the post‐metastatic intratumoral heterogeneity, combined with the apparent individual heterogeneity of the tumor, the choice of individualized chemotherapy drugs remains an intractable problem. Efficient and accurate drug screening models remain lacking in clinical practice.

Patient‐derived xenograft (PDX) models have been identified as a superior model system for translational research, as they maintain the characteristics of the original heterogeneous patient tumor.^[^ ^15^ ^]^ PDX models have been shown to be useful for predicting drug sensitivity or resistance of tumors to improve guidance for therapies for patients.^[^ ^16^, ^17^ ^]^ However, PDX is time‐consuming, has a low success rate, and requires many resources. In recent years, the patient‐derived tumor organoid model (PDTO) has been extensively studied as a preclinical cancer model for drug screening.^[^ ^18^, ^19^, ^20^ ^]^ Yao et al. demonstrated that organoids derived from patients with advanced rectal cancer can be used reliably as predictive models of response to chemoradiation and could potentially be used as a companion tool for rectal cancer treatment.^[^ ^21^ ^]^ In addition, Ganesh et al. indicated that PDTO could serve as a reliable tool for predicting chemoradiation response by establishing a murine endoluminal rectal cancer model.^[^ ^22^ ^]^ These studies have proven the physiological and predictive superiority of the PDTO platform over conventional methods, such as monolayer cell culture and PDX. Despite this, major problems with PDTO, which include the lack of standardized methods, complexity of organoid cultures, and reproducibility of manipulation, cannot be ignored.^[^ ^23^, ^24^ ^]^

In addition to these popular models, 3D‐bioprinting (3DP) models have become a novel biotechnique implicated by many emerging studies, which can rapidly establish in vitro “organs” with complex 3D architecture by using living cells in bioink and can simulate the microenvironment in vivo.^[^ ^25^, ^26^ ^]^ Furthermore, complex multicellular bioprinting is possible.^[^ ^27^, ^28^ ^]^ This frontier biotechnology has been applied to reconstruct several tissues and organs in vitro, such as the heart,^[^ ^29^ ^]^ vessels,^[^ ^30^ ^]^ skin,^[^ ^31^ ^]^ and bone.^[^ ^32^ ^]^ We previously successfully established a hepatorganoid model of HepRG cells using 3DP technology and demonstrated that transplanting 3DP hepatorganoids could prolong the survival of mice with liver failure. ^[^ ^33^ ^]^ In cancer research, cancer models established by the 3DP biotechnique represent a substantial improvement over traditional 2D models, which can restore the tumor microenvironment by mimicking space complexity and facilitating physiologically relevant cell‐cell and cell‐bioink interactions.^[^ ^34^ ^]^ 3DP tumor models have been applied in disease modeling and drug screening in a number of studies.^[^ ^35^, ^36^, ^37^ ^]^ We previously established a 3DP model of hepatocellular carcinoma cell lines that made drug screening possible.^[^ ^38^ ^]^ We also utilized patient‐derived tumor cells to print a 3DP tumor model and demonstrated that the 3DP tumor model could be applied in individualized therapy as a potential clinical tool.^[^ ^39^, ^40^ ^]^

In this study, we applied 3DP technology to establish patient‐derived CRC and CRLM models and demonstrated that 3DP tumor models maintained the specific biomarkers and characteristic mutation profiles of their parent tumors. Drug testing revealed marked tumor heterogeneity, including inter‐tumor heterogeneity among different patients and heterogeneity between primary and paired metastatic tumors within the same patient. We correlated the drug response data of CRLM 3DP models with patients’ clinical outcomes after neoadjuvant chemotherapy (NAC) and demonstrated that the 3DP cancer model could be a reliable and highly efficient platform for the individualized treatment of cancer.

## 2 Results

### 2.1 Construction of CRC Patient‐Derived 3DP Model

**Figure 1:**
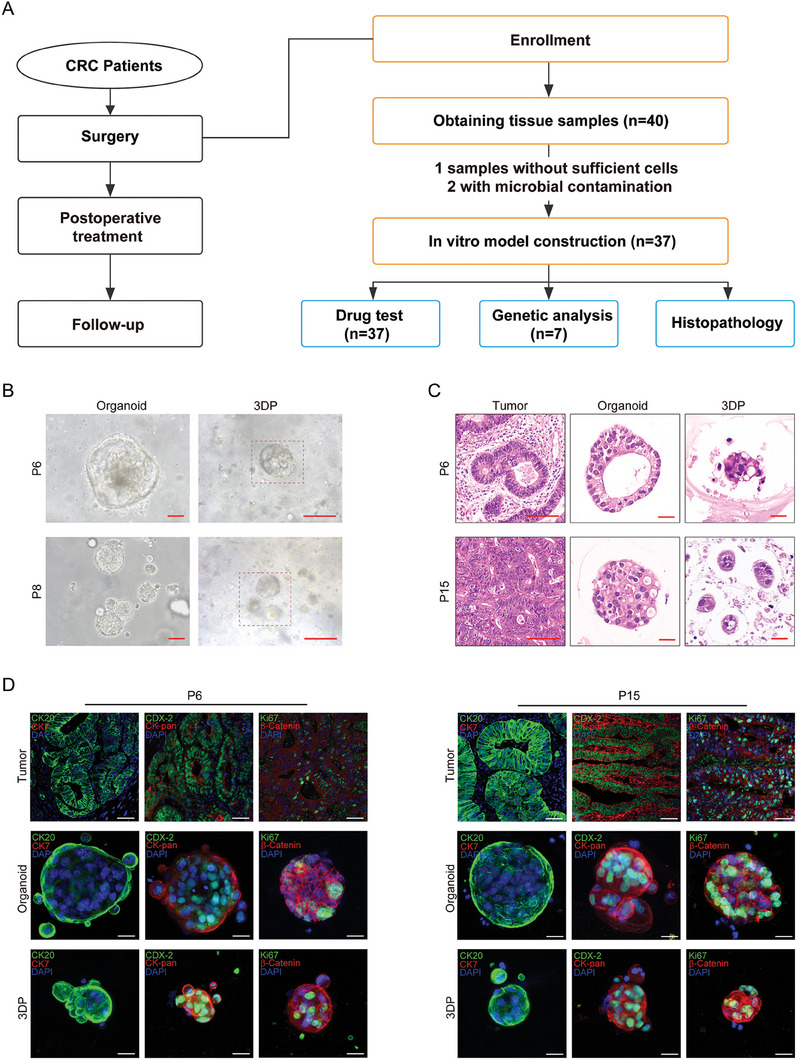
Study process and histopathological characterization of CRC 3DP models. A) The study process in primary colorectal cancer. A total of 40 patients undergoing surgery for CRC were enrolled, and 3DP models were successfully established and stably cultured for 37 patients. B) Bright‐field images of CRC 3DP models and CRC PDTOs on day 6 after production. Scale bar = 100 µm. C) HE staining comparing CRC 3DP models with corresponding organoids and parental tumors. Scale bar of tumor, 100 µm. Organoid and 3DP scale bars, 20 µm. D) CRC 3DP models and corresponding organoids and parent tumors were co‐stained with CK20 (green), CK7 (red), CDX2 (green), CK‐pan (red), β‐catenin (red), Ki‐67 (green) to examine the profiles of CRC biomarkers. DAPI was used to visualize nuclei (blue). Scale bar of tumor staining = 50 µm. Scale bars of 3DP and organoids staining = 20 µm.

This study enrolled 40 patients undergoing surgery for CRC in a clinical trial (ClinicalTrials.gov identifier NCT04755907), with informed patient consent (**Figure** **1**A). Patients’ baseline characteristics are shown in Table S1 (Supporting Information). All tumors were adenocarcinomas, based on pathology.

CRC 3DP models of 37 specimens were established and stably cultured. Two specimens could not be successfully established due to microbial contamination. One specimen was insufficient for cell extraction. Due to specimen size limitations, PDTOs were established in seven of 37 cases, passaged in Matrigel, and cultured in organoid medium, as previously described. Additionally, due to the limitation of specimen sizes, not all CRC 3DP models underwent primary cell extraction, whole‐exome sequencing (WES), and histopathological analysis. Specimens from seven patients underwent WES, and 37 underwent drug tests (Figure 1A).

Tumor cells were isolated as described previously and resuspended in gelatin (Gel)‐sodium alginate (SA) bioink for printing. The entire printing process was conducted inside the modeling chamber of the high‐precision 3D cell printer (SPP1603, SUNP) under a controlled temperature and sterile environment. The 6 mm × 6 mm × 1.2 mm grid‐like 3D structures obtained are shown in Figure S1A (Supporting Information). Primary CRC cells demonstrated a significant proliferative capacity, with over 85% of the cells maintaining viability in the 3DP models for a period of 2 weeks (Figure S1B,C, Supporting Information).

### 2.2 Histological and Genomic Features of CRC 3DP Models

The Gel‐SA composite bioink system is one of the most widely used low‐cost natural biomaterials in the field of micro‐extrusion‐based 3D bioprinting.^[^ ^41^, ^42^ ^]^ Notably, it boasts high grid porosity and remarkable cell compatibility. Through calcium ion crosslinking, it acquires controlled mechanical properties to prevent cell settling.^[^ ^43^, ^44^ ^]^ Numerous studies, including our previous research, have extensively validated the exceptional biocompatibility and highly controllable printability of this ink.^[^ ^33^, ^40^, ^45^, ^46^ ^]^ Within this context, we observed that primary tumor cells derived from CRC patients could proliferate into a diverse and irregular 3D sphere morphology within the porous microenvironment of the Gel‐SA bioink, akin to the behavior of tumor organoids (tumoroids) in PDTO, which similarly display a spontaneous assembly into various morphologies (Figure 1B; Figure S2A, Supporting Information; and Supplemental Videos). Significantly, the tumoroid‐like structures within the 3DP model exhibited a relatively consistent size within the porous Gel‐SA bioink, a stark contrast to the organoids in PDTO that showcased substantial variations (Figure S2B, Supporting Information).

Hematoxylin and eosin (HE) staining unveiled that certain tumoroid‐like structures within the 3DP model formed 3D structures with enclosed cavities or solid forms (Figure 1C). This architectural feature potentially resembles the glandular cavity‐like structures observed in some parental tumors (Figure 1C). PDTO specimens demonstrated significantly greater morphological diversity, a finding in line with prior research,^[^ ^21^ ^]^ including solid, thin cyst wall, and thick cyst wall structures (Figure 1B,C; Figure S2A, Supporting Information). Similar to CRC PDTO,^[^ ^21^, ^22^ ^]^ CRC 3DP models also could retain the biomarkers of the primary tumors from which they were derived. Biomarker expression analysis by immunofluorescence revealed that biomarkers detected in CRC tumors including CK7, CDX2, β‐catenin, Ki‐67, CK20, and pan‐cytokeratin (CK‐pan) were all significantly expressed in both CRC PDTOs and CRC 3DP models (Figure 1D; Figure S2C, Supporting Information). These similarities in biomarker staining patterns suggest that the 3DP models and organoids both could retain the specific histopathological features of the parental tumors.

**Figure 2:**
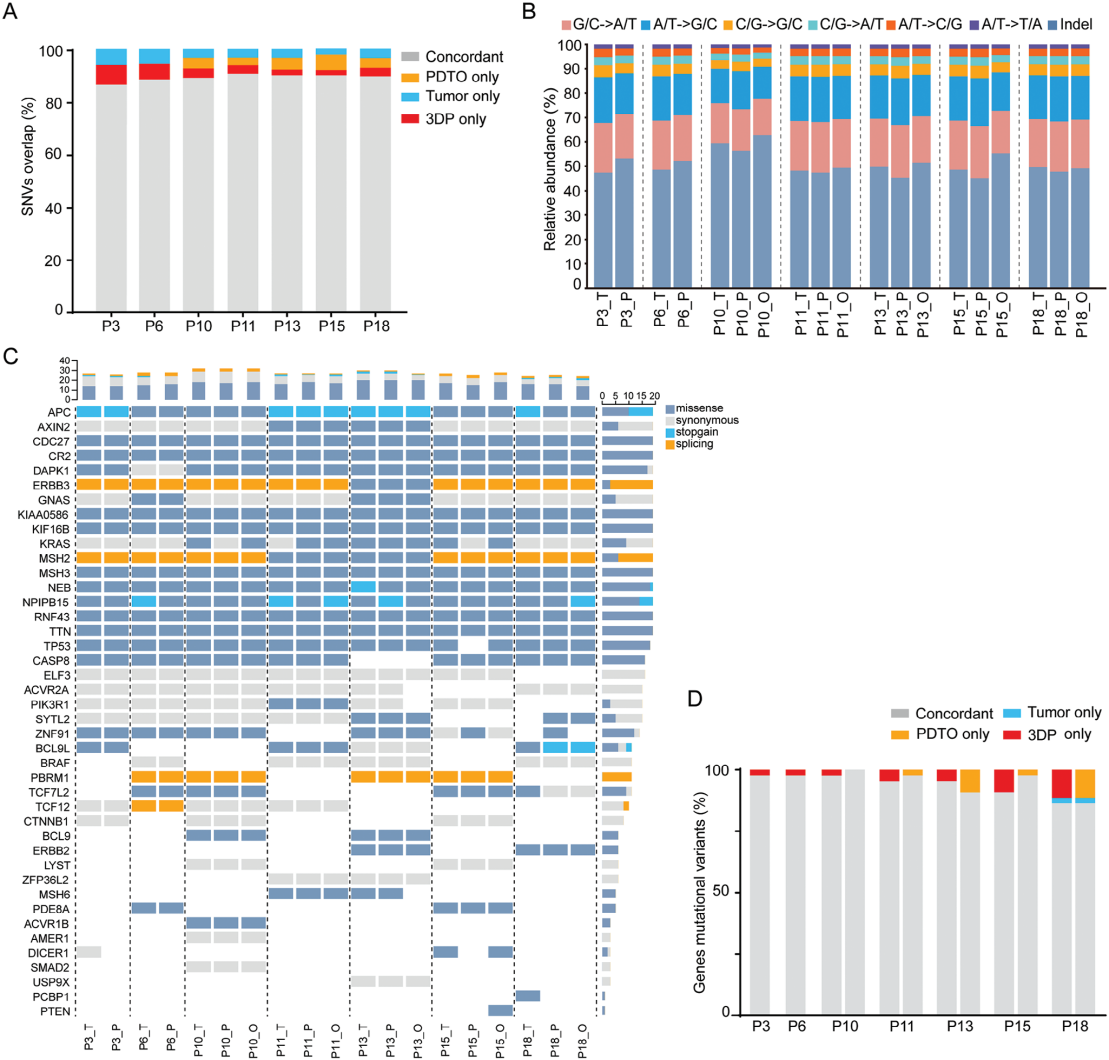
Genetic alteration profiles of CRC 3DP models and corresponding primary tumors. A) The concordance of single‐nucleotide variants (SNVs) of CRC 3DP models and CRC PDTOs with original tumor tissues. B) Proportions of exonic variants observed in all tested samples (T, tumor; P, 3DP; O, PDTO). C) Spectrum of SNVs in the most frequently mutated genes of CRC. Each row represented a driver gene, and each column represented the mutational profile of CRC 3DP models, CRC PDTOs, and parental tumors (T, tumor; P, 3DP; O, PDTO).

In previous studies, we revealed that 3DP tumor models derived from cancer patients recapitulate the genomic mutation profiles of corresponding tumors.^[^ ^40^ ^]^ To demonstrate that 3DP models derived from patients with CRC recapitulated the genomic profiles of the paired tumor tissues, seven 3DP models (P3, P6, P10, P11, P13, P15, and P18) at day 10 post‐bioprinting were subjected to WES along with their corresponding tumor tissues. As previous studies have revealed that PDTO maintains the genomic alterations of corresponding tumors.^[^ ^21^, ^47^, ^48^ ^]^ WES was also conducted on five PDTOs derived from P10, P11, P13, P15, and P18. Analysis of the results showed that the concordance of single nucleotide variations (SNVs) was well retained by the 3DP models with original tumor tissues for all tested specimens (**Figure** **2**A). Analysis of the proportion of SNVs and insertions/deletions (indels) within each group further demonstrated mutual similarity (Figure 2B). In addition, the concordance of exonic variants between the 3DP models and paired tumor tissues was comparable to that between the PTDO models and corresponding tumor tissues (Figure 2B).

More than 60 significantly mutated genes in CRC were selected from a recent study involving a large CRC cohort in the Chinese population^[^ ^49^ ^]^ and other previous studies.^[^ ^50^, ^51^, ^52^ ^]^ SNVs of these significantly mutated genes were analyzed, and mutations observed in at least one sample were shown (Figure 2C). The results illustrated that the CRC 3DP models retained the SNV spectrum of the significantly mutated genes in the original tumor tissues, although a few gains or losses were observed. Gene mutations associated with the Wnt signaling pathway were consistently observed in the 3DP model and PDTO of all seven specimens, and the mutation rate of APC was 100%. Significantly, in our results, seven CRC 3PD models retained a 94.2% overlap of the most frequent CRC gene mutational variants and their matching PDTOs also reached a high overlap rate (94.3%) (Figure 2D).

### 2.3 Chemotherapy Drugs Test of CRC 3DP Model

**Figure 3:**
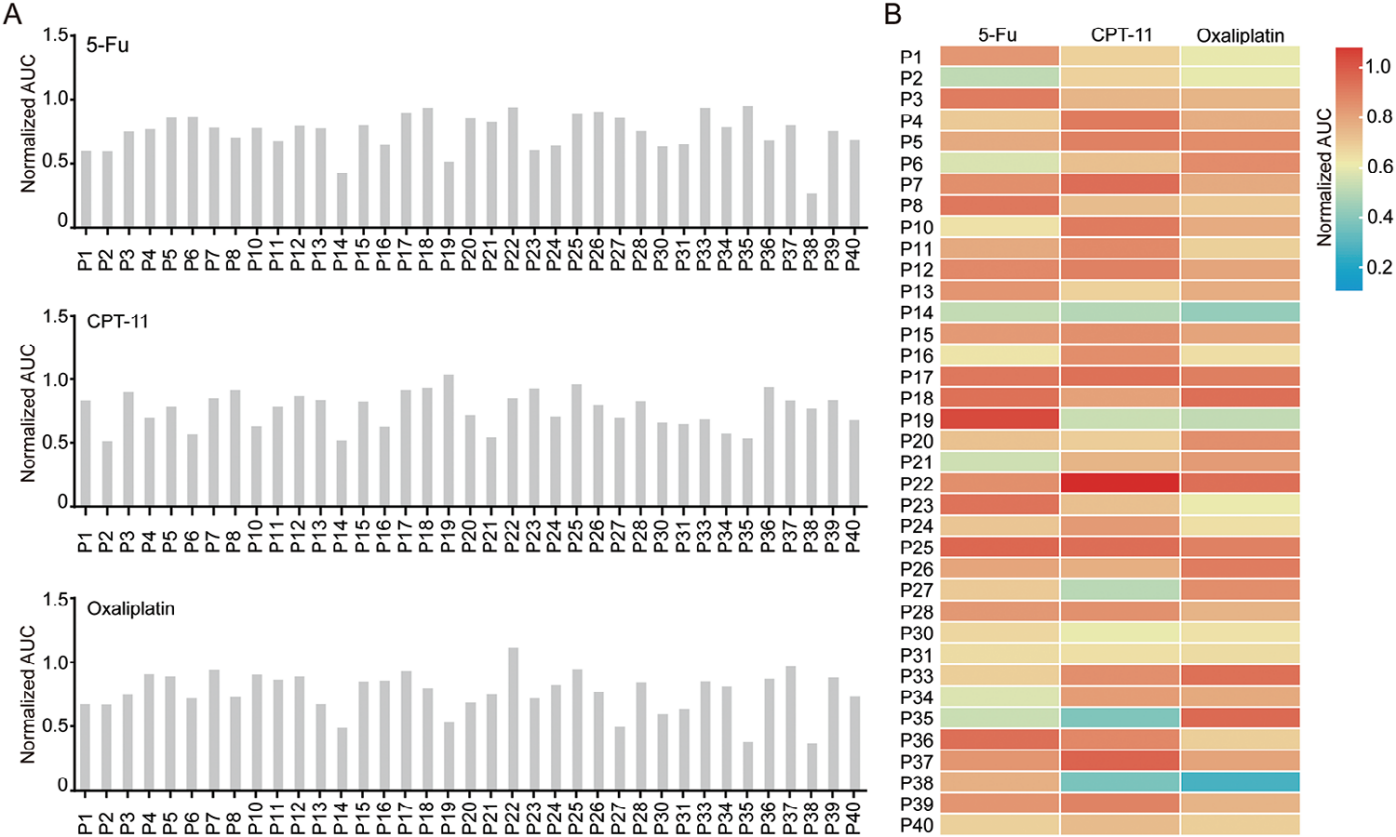
Drug responses of CRC 3DP models. A) The sensitivity of 37 CRC 3DP models to the chemotherapeutic drugs 5‐fluorouracil (5‐FU), irinotecan (CPT‐11), and oxaliplatin was assessed by determining the normalized area under the curve (AUC) from the corresponding dose‐response curves. B) Heatmap showing drug responses in CRC 3DP models of 37 patients. The responses to drug tests were presented as the AUC.

5‐FU, CPT‐11, and oxaliplatin are first‐line adjuvant chemotherapy drugs for CRC. We individually treated CRLM 3DP models in vitro with 5‐FU, CPT‐11, or oxaliplatin. We found that the relative cell viability of the CRC 3DP models was significantly different at 72 h after dosing (Figure S3, Supporting Information). The single chemotherapy agent sensitivity of the 37 CRC 3DP models to 5‐FU, CPT‐11, and oxaliplatin was presented by a normalized area under the dose‐response curve (AUC) calculated from each dose‐response curve (**Figure** **3**A). CRC 3D models derived from patients exhibited significant heterogeneity in their responses to single drug stimulation of 5‐FU, CPT‐11, or oxaliplatin, including heterogeneous responses of the same model to different drugs and heterogeneity across different models to the same drug. Such significant heterogeneity is represented by the wide range of normalized AUC values (0.2‐1.0) depicted in the heatmaps (Figure 3B).

### 2.4 CRLM Patient‐Derived 3DP Model Establishment and Development

**Figure 4:**
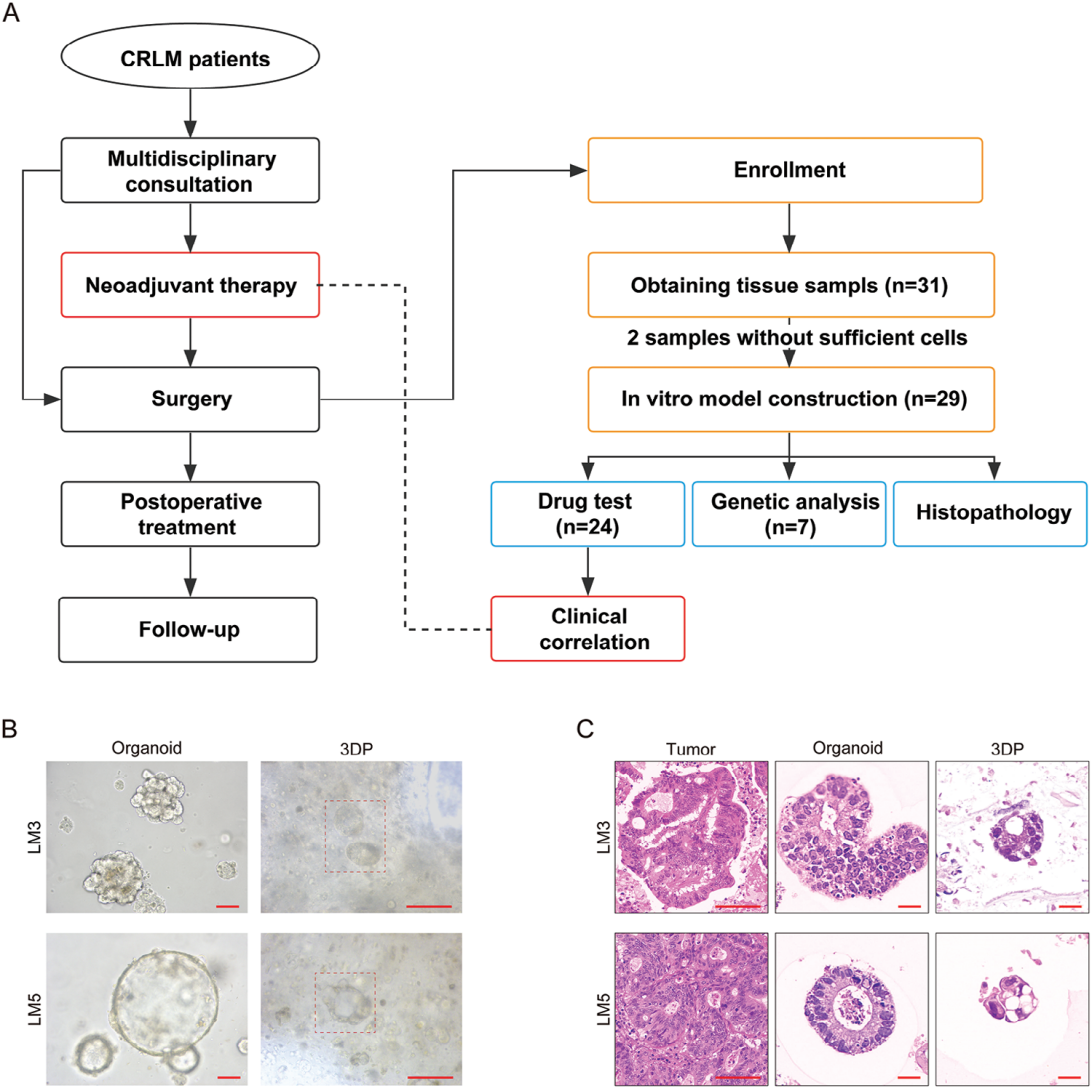
Establishment of the CRLM 3DP model. A) Study process in liver metastases. 31 patients undergoing CRLM surgery were enrolled. CRLM 3DP models of the 29 specimens were established. Drug responses were tested in 24 3DP CRLM models, among which 20 patients received neoadjuvant chemotherapy. B) Bright‐field images of CRLM 3DP models and PDTOs. Scale bar = 100 µm. C) HE staining of CRLM 3DP models, corresponding organoids, and parental tumors. Scale bar of tumor, 100 µm. Organoid and 3DP scale bars, 20 µm.

We further included 31 patients who underwent CRLM surgery in the aforementioned clinical trial, with informed patient consent (**Figure** **4**A); their baseline characteristics are shown in **Table** **S2** (Supporting Information). The pathological type of all the tumors was adenocarcinoma.

Tumor cells were isolated as described previously and were resuspended in Gel‐SA bio ink for printing. CRLM 3DP models of the 29 specimens were established and stably cultured. Simultaneously, eight PDTOs were successfully established and passaged in Matrigel. The main reason for the failure of the two specimens was insufficient tumor cell extraction. Unlike the CRC specimens, none of the CRLM specimens were contaminated with microorganisms. The cell viability assay demonstrated that primary CRLM cells remained viable in the 3D‐printed model over 2 weeks (Figure S4A, Supporting Information). Live and dead cell staining assays showed that most primary tumor cells (over 85%) remained alive within 2 weeks in 3DP models (Figure S4B, Supporting Information).

**Figure 5:**
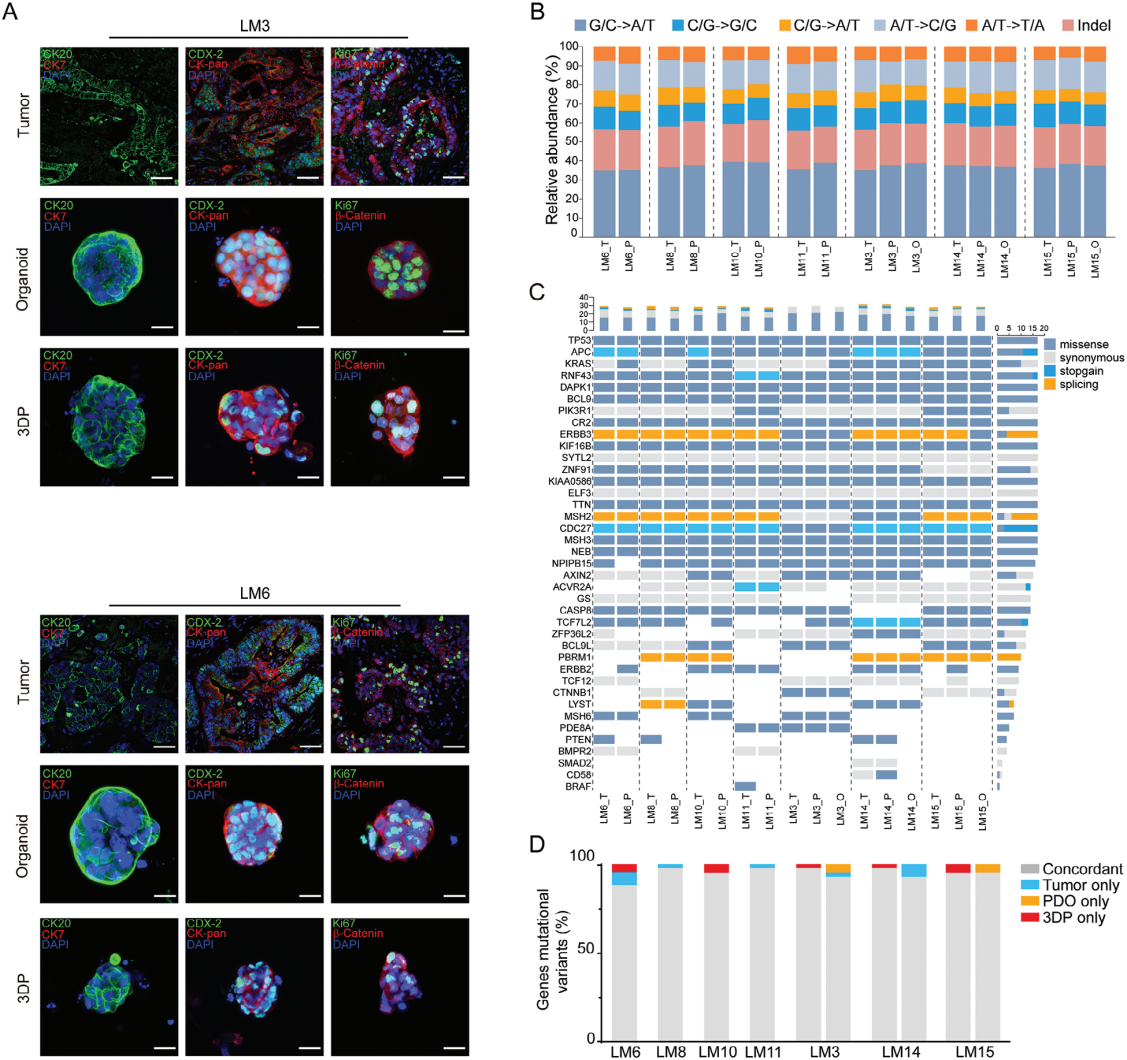
Histological and genetic mutational characterization of CRLM 3DP models. A) CRLM 3DP models and corresponding organoids and parent tumors were co‐stained with CK20 (green), CK7 (red), CDX2 (green), CK‐pan (red), β‐catenin (red), Ki‐67(green) to examine the profiles of CRC biomarkers. DAPI was used to visualize nuclei (blue). Scale bar of tumor staining = 50 µm. Scale bars of 3DP and organoids staining = 20 µm. B) Proportions of exonic variants for all tested samples (T, tumor; P, 3DP; O, PDTO). C) Spectrum of SNVs in the most frequently mutated genes of CRLM. Each row represents a driver gene, and each column represents the mutational profile of CRLM 3DP models, PDTOs, and parental tumors (T, tumor; P, 3DP; O, PDTO). D) Bar plots present the concordance among the SNVs of the most frequently mutated genes identified in CRLM 3DP models, PDTOs, and corresponding primary tumors.

Bright‐field images and HE staining revealed that primary CRLM cells formed tumoroid‐like structures in the CRLM 3DP model, exhibiting similar morphological features to the CRC 3DP model. In comparison with CRLM PDTOs, significant differences were observed in terms of morphology (Figure 4B,C; Figure S5A, Supporting Information). Much like the tumoroid‐like sphere sizes observed in the CRC 3DP model, the CRLM 3DP models also displayed remarkable consistency. Over a short period, these models exhibited relatively minor variations in cell sphere sizes, with the majority falling within the range of approximately 50 to 100 µm (Figure S5B, Supporting Information). Additionally, the CRLM 3DP models also retained the biomarkers of the primary tumors from which they were derived. Immunofluorescence biomarker expression analysis revealed that 3DP CRLM models, CRLM organoids, and paired parental tumors exhibited similar staining patterns for CK7, CDX2, β‐catenin, Ki‐67, CK20, and CK‐pan (**Figure** **5**A; Figure S5C, Supporting Information). The fluorescence expression of Ki67 in PDTOs was more prominent compared to that in the corresponding tumor tissues and 3DP models.

Similarly, to demonstrate that 3DP models derived from patients with CRLM recapitulated the genomic profiles of the paired tumor tissues, seven CRLM 3DP models (LM3, LM6, LM8, LM10, LM11, LM14, and LM15) in long‐term culture were subjected to WES, along with their corresponding tumor tissues. Analysis of the proportion of SNVs and indels indicated that the mutations in the original tumor tissues were retained by the CRLM 3DP models, and the concordance of mutations between 3DP models and the paired tumor tissues was comparable to that between PTDOs and the corresponding tumor tissues (Figure 5B).

Then, SNVs of significantly mutated genes of CRLM were analyzed using the above‐selected significantly mutated CRC genes. The results illustrated that the CRLM 3DP models also retained the SNV spectrum in the significantly mutated genes in the original tumor tissues, despite that there existed a few gains or losses of SNVs. (Figure 5C). Consistent with the analysis results of the CRC 3DP models, CRLM 3DP models retained a high (95.2%; 93.2% for CRLM PDTOs) overlap with the most frequent mutational variants in the parent tumor (Figure 5D). In addition, the expected levels of mutations in the WNT pathway were observed in CRLM 3DP model and PDTO cultures.

### 2.5 Responses of CRLM 3DP Models to Chemical Drugs that Correlate with Clinical Responses

**Figure 6:**
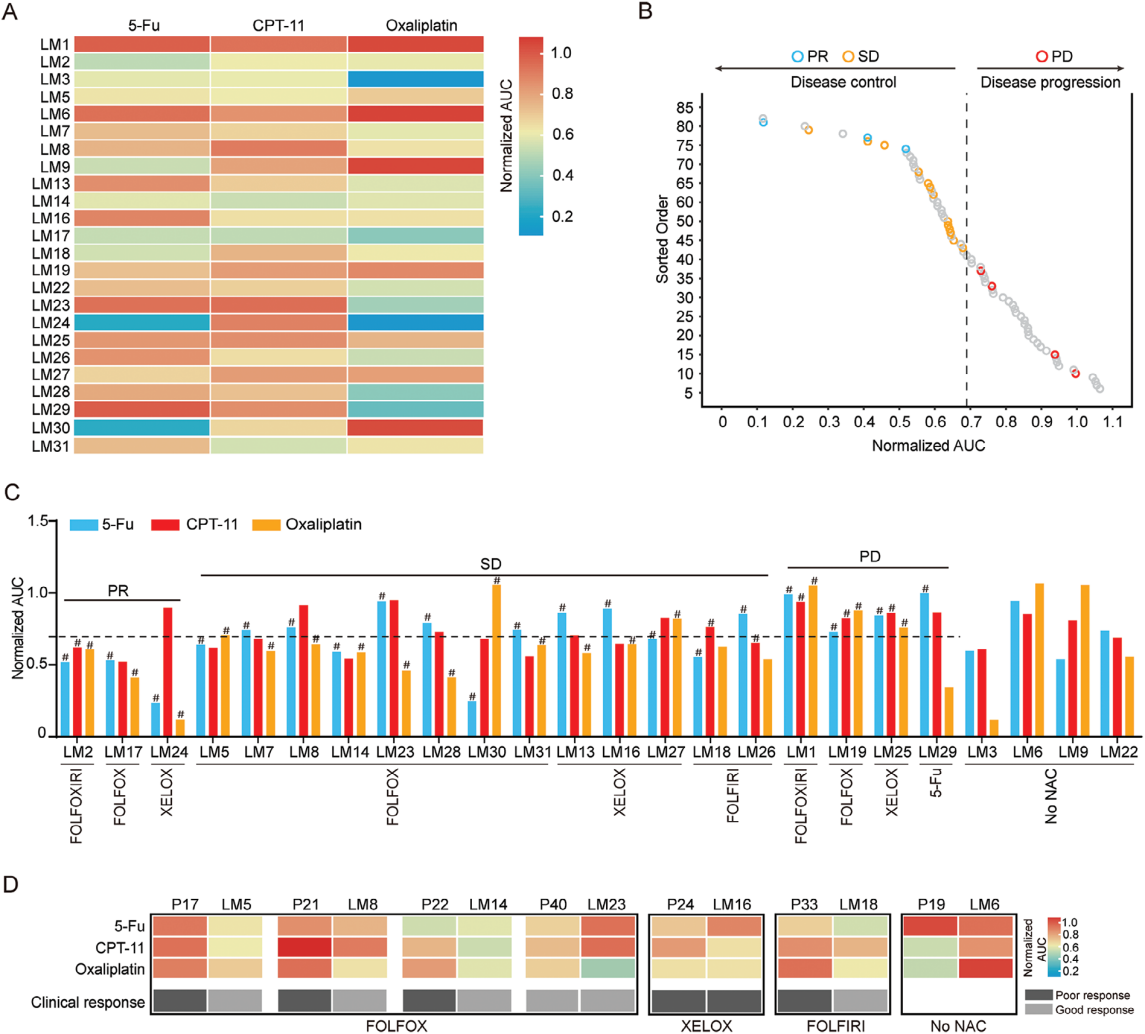
Correlation of CRLM 3DP models drug responses to clinical responses of neoadjuvant therapy. A) Heatmap showing drug responses in CRLM 3DP models of 24 patients. The responses to drug tests were presented as the normalized area under AUC. B) Correlation between normalized AUC values of drug tests and disease control of CRLM patients. All the normalized AUC values were shown as circles and ranked from the highest to the lowest. For each patient who received neoadjuvant chemotherapy, only the lowest AUC value of drugs used in the clinical regimen was highlighted, with the clinical response superimposed on the corresponding circle. Blue, yellow, and red denote PR, SD, and PD, respectively. The dotted line represents the cutoff value (0.690) of the normalized AUC values, which was generated by the Jenks natural breaks algorithm, classifying the CRLM 3DP models into sensitive or resistant to the drug. C) The normalized AUC values of each patient with known clinical response to neoadjuvant therapy. The dotted line symbolizes the cutoff value (0.690) for the classification of normalized AUC values. The pound sign represented the drug used in the patients’ chemotherapy regimens, and the regimens in clinic were shown at the bottom. D) Heatmap presents different drug responses and clinic outcomes between CRC primary lesions and their paired liver metastases.

Furthermore, drug sensitivity assays were performed on CRLM 3DP models from 24 patients to assess their response to 5‐FU, CPT‐11, and oxaliplatin. The normalized AUC, calculated from the dose‐response curves (Figures S6 and S7A, Supporting Information), was used to quantify the sensitivity of these models to each chemotherapy agent. Moreover, the heatmap analysis (**Figure** **6**A) reveals significant heterogeneity in the response of different CRLM 3DP models to various chemotherapy agents as well. In a clinical context, NAC was administered to 20 patients with CRLM. Ten patients underwent FOLFOX (chemotherapy regimen of 5‐FU, and oxaliplatin) regimen chemotherapy; five patients received XELOX (chemotherapy regimen of capecitabine (5‐FU) and oxaliplatin) regimen; two patients underwent pre‐treatment with FOLFIRI (chemotherapy regimen of 5‐FU, CPT‐11, and leucovorin calcium), and two patients underwent pre‐treatment with FOLFOXIRI (chemotherapy regimen of 5‐FU, CPT‐11, oxaliplatin and leucovorin calcium). One patient (LM29) who had undergone curative resection for primary colorectal cancer, presented with hepatic metastasis upon post‐treatment evaluation following a complete course of adjuvant monotherapy with 5‐FU. This case is categorized as a CRLM patient who received preoperative adjuvant monotherapy with 5‐FU.

To investigate the correlation between the results of in vitro drug sensitivity tests and the responses of CRLM patients to NAC, we ranked all the normalized AUC values of the drug tests from the highest to the lowest (Figure 6B). For each patient who received NAC, the lowest normalized AUC values of the drugs used in the treatment were highlighted with annotations indicating clinical outcomes. As shown in Figure 6B, there were no overlaps among the lowest AUC values of patients considered to show disease control (partial response, PR, and stable disease, SD) and disease progression (PD) in the clinic. Furthermore, we utilized the Jenks Natural Breaks algorithm to classify data points into groups. To avoid overfitting, the classification that generated the minimum number of breaks to obtain a goodness‐of‐fit greater than 90% was adopted (Figure S7B, Supporting Information). Based on this, all normalized AUC values were segregated into four groups. A Jenks break of 0.690 allowed the classification of normalized AUC values for discriminating the 3DP models as sensitive or resistant in drug tests. If any drug used in the treatment generated a normalized AUC value lower than 0.690, the 3DP model was regarded as sensitive to that regimen. 3DP models were designated as resistant to the clinical regimen only if all drugs yielded a normalized AUC higher than 0.690 (Figure 6B).

For instance, the CRLM 3DP models from LM1, LM19, and LM25 were all triple‐resistant, and the patients had a poor response. LM29 demonstrated resistance to 5‐FU and CPT‐11 while exhibiting sensitivity to oxaliplatin. However, the patient underwent adjuvant treatment with 5‐FU monotherapy, leading to the rapid emergence of hepatic metastases. CRLM 3DP models from LM2 and LM17 were triple‐sensitive, and the two patients achieved a good clinical response. Patient LM24 displayed sensitivity to both medications (5‐FU and oxaliplatin) included in the treatment regimen (XELOX) and achieved a favorable clinical response.

The 3DP models derived from patients who achieved a stable clinical response demonstrated sensitivity to at least one of the medications included in the treatment regimen. Figure 6C summarizes the patients’ treatment regimens, clinical outcomes, and CRLM 3DP model responses to drug tests. In our findings, patients with CRLM who did not show disease progression were sensitive to at least one chemotherapeutic agent in the chemotherapy regimen they received. These results demonstrated that the responses of the 3DP CRLM model to the drug tests matched well with the clinical outcomes of the corresponding patients.

### 2.6 Different Drug Responses Between Primary Colorectal Cancer and its Metastases

Among the CRC and CRLM patients included in this study, we obtained primary and metastatic specimens from seven CRC patients with synchronous liver metastases (P17/LM5, P19/LM6, P21/LM8, P22/LM14, P24/LM16, P33/LM18, and P40/LM23). As presented in Figure 6D, one patient (P19/LM6) did not receive NAC, one patient (P24/LM16) received the XELOX regimen, one patient (P33/LM18) received the FOLFIRI regimen, and other four patients (P17/LM5, P21/LM8, P2/LM14, and P40 /LM23) were treated with the FOLFOX regimen. The responses of these seven pairs of tumors 3DP models in drug tests demonstrated that the drug response of the primary CRC tumors and liver metastases show marked heterogeneity between primary and metastases in the same chemotherapy regimen, even to a single agent in the regimen (Figure 6D).

## 3 Discussion

The heterogeneity of patients with advanced cancer is considered to be the principal reason for the failure of cancer drug therapies.^[^ ^14^ ^]^ The selection of sensitive drugs among the many existing treatment options for individualized treatment is a challenge for oncologists. With the development of 3DP technology, the establishment of a tumor model by 3DP holds great promise in cancer research, particularly for personalized cancer treatment.^[^ ^34^, ^53^ ^]^ To date, relevant research in oral cancer,^[^ ^54^ ^]^ breast cancer,^[^ ^55^ ^]^ colorectal cancer,^[^ ^56^ ^]^ and lung cancer^[^ ^57^ ^]^ has shown that 3DP technology can be a novel platform to study tumor development and shows the potential to accelerate cancer research dramatically.^[^ ^34^, ^35^, ^36^ ^]^

In contrast to immortalized cancer cell lines, individualized patient‐derived tumor cells have been broadly employed in high‐throughput screening of drug candidates and cancer biomarkers, because they retain tumor heterogeneity and genomic features.^[^ ^58^ ^]^ Efficient, rapid, and practical generation of 3D models is imperative when handling freshly isolated patient‐derived tumor cells. We employed an automated 3D bioprinting platform that successfully executed this strategy. We have further enhanced the optimization of a proven Gel‐SA bioink‐based 3D model system by designing a refined, intricate, mesh‐like 3D structure using a computer platform (Figure S1A, Supporting Information). This has enabled us to efficiently create sufficiently homogenized 3D tumor models in a short duration. Herein, we employed a high‐precision bioprinter to successfully establish patient‐derived personalized models for both CRC and CRLM. Notably, this marks the pioneering achievement of establishing patient‐derived CRC/CRLM tumor models through 3D biotechnology, achieving a remarkable success rate of 93.0%. The entire workflow, commencing from the surgical procedure to the bioprinting of the 3DP tumor model, takes less than 2 hours. Our findings underscore the good viability of primary tumor cells within the 3D bioink configuration and their excellent activity prior to commencing in vitro treatments.

Our investigation revealed that primary tumor cells within the 3DP models have the capacity to generate 3D structures, encompassing both enclosed cavities and solid formations (Figures 1C and 4C). This phenomenon potentially corresponds to the histomorphological traits of the parental tumor; however, a broader sample size is warranted to firmly establish this connection. Due to the excellent printability of this specific bioink and the precision of the bioprinter, the tumor volume in 3DP models exhibits minimal variation and a uniform distribution, resulting in outstanding reproducibility. In contrast to PDTO, which exhibits substantial variability in construction methods and organoid sizes, and lacks standardized protocols, resulting in significant labor and material expenses,^[^ ^23^, ^24^ ^]^ the 3DP tumor model offers distinct advantages. These include its cost‐effectiveness, efficient modeling process, higher success rate, ability to maintain cell viability, and reduced time requirements for procedures. Moreover, 3DP technology can be utilized to create diverse cell models that accurately assemble the immune microenvironment in vitro, thereby making the 3DP tumor model even more promising for individualized drug prediction and cancer research.

In our study, the establishment of 3DP tumor models requires a substantial quantity of primary tumor cells. This aligns with the cellular density prerequisites for constructing 3D biofabricated tissue engineering models (round from 1 to 10 million cells/ml).^[^ ^59^, ^60^ ^]^ Utilizing micro‐extrusion‐based bioprinting allows for the rapid creation of bio‐models containing high‐density cells that can function effectively within a short timeframe, closely mimicking native tissues. Additionally, both excessively low and high cellular densities can affect the physicochemical properties of the bioink.^[^ ^43^, ^61^, ^62^ ^]^ Specifically, for our investigation involving 3D bioprinting primary tumor cells sourced from patients, maintaining a concentration of 3 to 5 million cells per milliliter in our bioink is appropriate. This concentration promotes the optimal growth status for primary tumor cells within a short time window, facilitating reliable and accurate drug testing outcomes. Presently, a primary limitation in achieving successful 3DP tumor establishment is the restricted yield of cells obtained from specimens. Consequently, we refrained from utilizing tumor specimens procured via biopsy forceps.

Our existing results illustrated that the patient‐derived tumor 3DP model can retain the biomarkers and genomic characterization of original tumors. Drug response results from 37 CRC 3DP models and 24 CRLM 3DP models illustrated notable inter‐patient heterogeneity of CRC and CRLM, which may be more pronounced than previously realized. The preservation of heterogeneity suggests that the 3DP models can be applied to predict individualized therapies.

Recently, Mo et al. established a living biobank with 50 organoids derived from paired CRC and CRLM lesions, with an overall success rate of 80.6%. Their results showed that CRLM PDTOs have excellent potential for predicting the chemosensitivity and clinical prognosis of patients.^[^ ^63^ ^]^ In our CRLM study, 20 patients with CRLM received NAC. Based on our analysis of the drug test results, we determined that the drug responses of the tested 3DP models had correlation with the clinical outcomes of the corresponding CRLM patients who underwent NAC. During the analysis, we adopted an approach from a recent study by classifying the AUC derived from the dose‐response curve,^[^ ^64^ ^]^ which was then translated into clinical information. The chosen predictor AUC avoided disregard of the potential clinical utility of chemotherapeutics as much as possible. We did not use combinations of drugs in the 3DP model because it was difficult to determine the combined drug concentration in vitro. The clinical response of most combination drug therapies can be explained by monotherapy,^[^ ^65^, ^66^ ^]^ however, the potential difference between combination therapy and independent single‐drug therapies in the 3DP tumor model deserves further study.

It is worth noting that the primary tumor and metastases in the same patient may respond differently to the same chemotherapy regimen. Mo et al. demonstrated that primary and metastatic lesions from the same patient were heterogeneous in molecular fingerprints, but the majority results of drug sensitivity in vitro were highly consistent. In our study, we obtained primary tumor and metastatic specimens from seven patients with synchronous liver metastases from CRC and performed the same drug response tests. Drug test results are expressed in the predictor AUC as previously described. We found that CRC patients with synchronous liver metastases demonstrated different responses to single chemotherapy drug between the primary tumors and metastases. This indicated tumor‐to‐tumor heterogeneity between the primary tumors and metastases which could not be ignored. We found that the primary and metastatic lesions of four patients who received NAC had different pathological regression responses. In our results, the metastatic lesions of these four patients all showed good pathological responses relatively, whereas the primary lesions did not respond considerably. These results illustrated that the existing heterogeneity in chemotherapy drug test results and clinical treatment responses between primary and metastatic lesions of part patients are important issues that should be taken into account in precision medicine. Due to the limitations of the specimen size, we were able to perform drug tests only for paired primary and metastatic lesions. WES was not performed to analyze the differential gene profiles that may exist between the primary and metastatic foci.

## 4 Conclusion

In conclusion, we have successfully established a 3DP tumor model for patients with CRC/CRLM, which, to our knowledge, has not been reported previously. We demonstrated that this model could maintain the biological and genetic features of the original tumor, and importantly, we found a significant correlation between clinical outcomes in patients with CRLM and individual 3DP tumor model drug sensitivity tests, considering the significant tumor heterogeneity. The next generation of individualized treatment will likely involve a more precise medicine model, which requires incorporating the heterogeneity of the patient population into every step of the diagnosis‐treatment cascade. While individualized treatment of advanced malignancies, including CRLM, is currently relatively ambiguous, our findings indicate that the patient‐derived cancer 3DP model has the potential to serve as a platform to enhance precision cancer treatment and expedite the clinical development of new drugs. However, given the relatively small sample size in our study, further studies are required to test this method in a larger patient population.

## 5 Experimental Section

### Participants and Specimens

Patients with CRC/CRLM were recruited from an observational clinical trial (ClinicalTrials.gov identifier: NCT04755907) in four medical centers: Peking Union Medical College Hospital, Cancer Hospital Chinese Academy of Medical Sciences, Beijing Cancer Hospital, and China‐Japan Friendship Hospital. The enrollment criteria included age ≥ 18 years, clinical diagnosis of CRC/CRLM, and feasibility of surgical treatment. CRC and CRLM were confirmed by postoperative pathological diagnosis in all participants. Written informed consent was obtained from all the patients prior to tissue acquisition. The study was conducted in accordance with recognized ethical guidelines and received approval from the Ethics Review Committee of Peking Union Medical College Hospital (Approval No. JS‐2475).

The specimens were transported on ice in DMEM/F12 (Gibco, Billings, MT, USA) medium with primocin (0.2%) (InvivoGen, San Diego, CA, USA), antibiotic‐antimycotic (1%) (Gibco), and RHOK inhibitor Y‐27632 (10 µm) (Sigma–Aldrich, St Louis, MO, USA), and arrived at the laboratory as soon as possible after surgery. Upon arrival, each specimen was swiftly divided into several parts according to size for primary cell extraction, WES, or histopathological analysis.

### Cell Isolation, Bioprinting, and Culture

Specimens for tumor cell isolation and culture were washed in cold phosphate buffered solution (PBS) with primocin (0.2%), antibiotic‐antimycotic (1%), and Y‐27632 (10 µm) for 5 min × 3 min to avoid contamination by microorganisms and were then minced into pieces smaller than 3 mm, on ice. Then, the specimen fragments, in cold PBS, were transferred to a 15 mL centrifuge tube and centrifuged at 100 × *g* for 3 min until the supernatant became clear. Minced tissues were incubated in digestion medium (DEME/F12 medium supplemented with collagenase II (1.5 mg mL^−1^) (Gibco), dispase type II (1 mg mL^−1^) (Sigma–Aldrich), hyaluronidase (20 µg mL^−1^) (Sigma–Aldrich), Y27632 (10 µm), antibiotic‐antimycotic (1%), and primocin(0.2%)) at 37 °C on an orbital shaker at 37 °C for 30–60 min and were shaken manually every 15 min. Tumor cells were collected after filtering through a 100‐µm cell strainer (Biosharp, San Diego, CA, USA), centrifuged at 300 × *g* for 5 min, and resuspended in 3DP culture medium after cell counting for bioprinting.

To formulate the bioink, Gel and SA were mixed evenly with the cell suspension in proportion: Briefly, bioink was mixed well with the cell suspension, 4% SA (Sigma–Aldrich), and 12% Gel (Sigma–Aldrich) at a volume ratio of 2:1:2, resulting in a final concentration of tumor cells of 5 × 10^6^ cells mL^−1^ and the final concentration of Gel and SA was 4.8% and 0.8%. The bioink cell/biomaterial mixture was drawn into a 3 mL syringe (BD, Franklin Lakes, NJ, USA) with a 23 G needle. After incubating at 4 °C for 30 min until ink gels were formed, the syringe was set into a 3D bioprinter (Cherry Hill, NJ, USA). The temperature of the nozzle and the chamber was set to 20 and 10 °C, respectively. The 3DP model was designed as layer‐by‐layer grids with a size of 6 mm × 6 mm × 1.2 mm (layer height was 0.2 mm and line width was 0.8 mm) and was printed using an extrusion speed of 1.5 mm^3^ s^−1^. The printing process was conducted within 2 h, during which the 3D bioprinting models were collected in 48‐well plates on the chamber platform.

Fresh 3DP grids were immersed in calcium chloride solution (3%) for 2 min to cross‐link the SA, thus providing better physical strength, and then washed with Hank's Balanced Salt Solution (HBSS) (Gibco). Each 3DP model was cultured in 500 µl 3DP medium, which was composed of advanced DMEM/F12 (Gibco), B27 supplement (1×) (Life Technologies, Carlsbad, CA, USA), N2 supplement (1×) (Life Technologies), GlutaMAX (2 mm) (Gibco), HEPES (10 mm) (Gibco), recombinant EGF (50 ng mL^−1^) (PeproTech, Rocky Hill, NJ, USA), N‐acetyl‐L‐cysteine(1 mm) (Sigma–Aldrich), Prostaglandin E2 (10 nm) (Sigma–Aldrich), A8301 (500 nm) (Tocris Bioscience, Bristol, UK), SB202190 (3 εm) (Sigma–Aldrich), Gastrin I (10 nm) (Sigma–Aldrich), primocin (0.2%), antibiotic‐antimycotic (1%), and Y27632 (10 µm). The 3DP medium was refreshed every 3 days. To assess the viability of the 3DP CRLM models, Calcein‐AM (CAM, Sigma–Aldrich) and propidium iodide (PI, Sigma–Aldrich) staining were performed on days 1, 3, 6, 10, and 14 after bioprinting. This staining method allows for the discrimination of viable cells, which were labeled green, from dead cells, which were labeled red. Additionally, the CellTiter‐Glo 3D Cell Viability Assay (Promega, Madison, WI, USA) was used to evaluate the cell viability of the CRC 3DP model.

To establish PDTOs, the tumor cell pellet was suspended in Matrigel and promptly inoculated into preheated 24‐well flat‐bottomed cell culture plates (Costar, Washington, DC, USA) in a dome shape, using 50 µL volume per well. After a 10‐min incubation at 37 °C in 5% CO_2_, the wells were covered with 500 µL of 3DP medium. PDTOs were maintained by refreshing the medium every 3 days, while monitoring and capturing images of the organoids at specific time points using a microscope. Organoid passaging was performed every 1–2 weeks based on their density. Initially, organoids were dissociated using TrypLETM Express (GIBCO) and mechanically sheared through bovine serum albumin‐coated (1%) pipette tips, followed by multiple centrifugation washes at 300 × *g* until the Matrigel was cleared. The organoid fragments were then resuspended in Matrigel and seeded as described above. Cryopreservation of organoids was conducted using a serum‐free medium (NCM Biotech, Jiangsu, China), and upon recovery, the medium was supplemented with Y27632 (10 mm) for culture.

### HE, and Immunofluorescence Staining

Fresh specimens were fixed with paraformaldehyde (PFA) (4%) (Sigma–Aldrich). PDTOs and 3DP models were collected and fixed in 10% formaldehyde calcium (Solarbio, Beijing, China) overnight after culturing for 10 days. After embedding in paraffin, specimens were sectioned into 5‐µm‐thick slices and subsequently stained with HE following standard procedures.

For immunofluorescence, fresh tumor specimens were fixed in PFA (4%) overnight, dehydrated using a gradient of sucrose solution, embedded in Tissue‐Tek O.C.T. compound (Sakura Finetek, Torrance, CA, USA), and cut into 5‐µm‐thick slices. PDTOs and 3DP models were harvested 10 days after manufacturing, dissociated gently, and seeded in chambered cell culture slides coated with Gel (3%), where they were cultured overnight in 3DP culture medium. They were handled using standard immunofluorescence procedures. Briefly, the tissue sections were fixed in 4% PFA for 15 min. Dissociated units of 3DP CRC/CRLM models and PDTOs on chambered cell culture slides were fixed in 10% formaldehyde for 15 min. Then, all samples were permeabilized with Triton X‐100 (0.3%) (Sigma–Aldrich) for 20 min at room temperature, blocked in 1% bovine serum albumin (Sigma–Aldrich) for 30 min at room temperature, and incubated with primary antibodies in blocking buffer at 4 °C overnight. Rabbit anti‐CK20 (1:200, Abcam, Cambridge, MA, USA), mouse anti‐CK7 (1:200, Abcam), rabbit anti‐CDX2 (1:200, Abcam), rabbit anti‐β‐catenin (1:200, Abcam), mouse anti‐Ki‐67 (1:200, Abcam), and mouse anti‐CK‐pan (1:200, Abcam) were used as primary antibodies for immunohistochemical staining. The 3DP models and sections were then incubated with goat anti‐rabbit IgG Alexa Fluor 488 (1:300, Abcam) and goat anti‐mouse IgG Alexa Fluor 594 (1:300, Abcam) for 2 h at room temperature. 4′,6‐Diamidino‐2‐phenylindole (DAPI) (20 µm, Sigma–Aldrich) was used to label the nuclei at room temperature. The stained sections were mounted in an anti‐fade solution containing DAPI (Abcam). The samples were then washed with HBSS. Stained cells and sections were observed under a laser scanning confocal microscope (Nikon A1, Tokyo, Japan).

### Whole‐Exome Sequencing and Mutation Analysis

DNA from CRC/CRLM specimens and corresponding CRC/CRLM 3DP models and PDTOs of patients was extracted using the TIANamp Genomic DNA Kit (Tiangen Biotech, Beijing, China) following the manufacturer's protocol after culturing for 10 days. Prior to library construction, the extracted DNA was strictly tested as follows: i) detection of DNA purity using a NanoPhotometer spectrophotometer (IMPLEN, Westlake Village, CA, USA); ii) quantification of DNA concentration using the Qubit DNA Assay Kit in a Qubit 2.0 Fluorometer (Life Technologies); iii) evaluation of DNA degradation and contamination on agarose gels (1%). The whole exome was captured using the Agilent SureSelect Human All Exon V6 platform (Agilent, Santa Clara, CA, USA) and sequencing was performed on an Illumina HiSeq 4000‐PE150 (Illumina, San Diego, CA, USA).

Reads were aligned to the human reference genome (GRCh37/HG19) using the Burrows‐Wheeler Aligner (BWA, version 0.7.9a).^[^ ^67^ ^]^ Local realignment and base quality score recalibration were performed using the Genome Analysis Toolkit (GATK),^[^ ^68^ ^]^ with duplicate reads removed using Picard. SNVs and indels were simultaneously called with the HaplotypeCaller of GATK (v3.3.0).

### Drug Tests and Cell Viability Assay

Drug treatment in the 3DP models was conducted on day 6, the time‐point of the second medium change. 5‐FU (Selleck), CPT‐11 (Selleck), and oxaliplatin (Selleck), three drugs commonly applied as chemotherapy of CRC/CRLM, were tested in the 3DP CRC/CRLM models, as single agents. Drugs were added to 500 µL culture medium according to a set concentration gradient (100, 50, 10, 1, and 0 µm), and the drug‐containing culture medium was refreshed every 24 h for 72 h. Cell viability was detected using the CellTiter‐Glo 3D Cell Viability Assay (Promega, Madison, WI, USA), according to the manufacturer's instructions.

### Clinical Information for Outcome Evaluation of Patients Who Underwent Chemotherapy Treatment

Patients received standard treatment according to the guidelines for CRC/CRLM after multidisciplinary consultation. Some participants who were potentially eligible for surgical treatment were recommended to receive neoadjuvant therapy before surgery, while others underwent surgery without NAC. The most widely used regimens of (neo) adjuvant chemotherapy for CRC and CRLM patients included FOLFOX, FOLFIRI, FOLFOXIRI, and XELOX.

Clinical responses to NAC for CRLM were evaluated based on radiological examinations by attending physicians, independent of the laboratory work. Chemotherapy responses of CRLM patients were classified as PR, SD, or PD used to evaluate CRLM tumor response in this study, according to the Response Evaluation Criteria in Solid Tumors (RECIST) version 1.1. PR and SD were both classified as disease control. Tumor and node staging for all resected specimens were conducted according to the 7th American Joint Committee on Cancer TNM staging manual.

For CRC, tumor regression grade (TRG) was mainly used to evaluate the clinical response of patients to NAC in this study. All tumor slides were examined at the microscope by independent pathologists. To compare tumor response between paired specimens from the same patient, tumor regression was scored in CRC after NAC using a modified Mandard protocol^[^ ^69^ ^]^ that can also be applied to CRLM.^[^ ^70^ ^]^ The modified Mandard protocol was based on the presence of residual tumor cells and the extent of fibrosis, which were defined as follows: TRG 1, complete regression with no residual tumor; TRG 2, presence of rare residual cancer cells; TRG 3, presence of larger numbers of residual cancer cells with predominant fibrosis; TRG 4, residual cancer outgrowing the fibrosis; and TRG 5, absence of regressive changes. TRG was associated with prognosis in patients receiving preoperative chemotherapy.^[^ ^70^ ^]^ Considering the degree of tumor regression and survival benefit of the patients, the patients were divided into two groups. Tumors with TRG 1, 2, or 3 tended to show a good response to NAC, and those with TRG 4 or 5 tended to show a poor response to NAC.

### Statistical Analysis

Cell viability of the 3DP models after drug treatment was normalized to the mean of the untreated controls. Dose‐response curves of drug testing were fitted using weighted n‐parameter logistic regression in the R package “nplr”.^[^ ^71^ ^]^ The AUC was inferred according to Simpson's rule, and the normalized AUC was obtained by dividing the corresponding AUC value by the maximum area for each concentration range.

To understand the correlation between the results of drug testing and the patients’ clinical responses, normalized AUC values of CRLM 3DP models were ordered and then clustered into groups according to the Jenks Natural Breaks algorithm.^[^ ^72^ ^]^ The number of breaks was defined as the minimum that reached a goodness‐of‐fit exceeding 90%. By minimizing intragroup variance and maximizing intergroup variance, this algorithm identified cutoff values to discriminate drug tests as sensitive or resistant in this study as well as in previous research.^[^ ^64^ ^]^ Relevant analyses between lab drug testing and the clinical responses were performed using the R package “jenks” in the “classInt”. All statistical analyses were conducted using R software version 4.0.1 (www.r‐project.org).

## Conflict of Interest

The authors declare no conflict of interest.

## Author Contributions

H.S., L.S., X.K., and L.L. contributed equally to this work. Y.M., H.Y., and P.H. conceived, designed, and supervised the study, obtained funding and provided administrative, technical, or material support. H.S., L.S., X.K., and L.L. contributed to the development of methodology. L.S., L.L., C.L., and B.J. contributed to the analysis and interpretation of data. H.S. and X.K. contributed to the writing of the manuscript. P.W., Z.J., H.Z., Z.Y., Y.S., J.L., Y.W., M.S., M.P., Y.H.W., B.W., H.T.Z., X.S., and B.X. contributed to the acquisition of data and material. Y.M., H.Y., P.H., H.T.Z., and X.S. contributed to the review and revision of the manuscript. All authors read and approved the final manuscript.

## Supporting information

Supporting Information

Supplemental Video 1

Supplemental Video 2

## Data Availability

The data that support the findings of this study are available from the corresponding author upon reasonable request.
