# Supplementary material for: Prediction of Clinical Precision Chemotherapy by Patient‐Derived 3D Bioprinting Models of Colorectal Cancer and Its Liver Metastases
Source: Adv Sci (Weinh). 2023 Nov 16;11(2):2304460. doi: 10.1002/advs.202304460 (PMC10787059; doi:10.1002/advs.202304460)
Supplement: Supplementary file 1 — Supporting Information [file ADVS-11-2304460-s001.pdf]

ADVANCED SCIENCE
Open Access

Supporting Information

for *Adv. Sci.*, DOI 10.1002/advs.202304460

Prediction of Clinical Precision Chemotherapy by Patient-Derived 3D Bioprinting Models of Colorectal Cancer and Its Liver Metastases

Hang Sun, Lejia Sun, Xindi Ke, Lijuan Liu, Changcan Li, Bao Jin, Peipei Wang, Zhuoran Jiang, Hong Zhao, Zhiying Yang, Yongliang Sun, Jianmei Liu, Yan Wang, Minghao Sun, Mingchang Pang, Yinhan Wang, Bin Wu, Haitao Zhao, Xinting Sang, Baocai Xing, Huayu Yang*, Pengyu Huang* and Yilei Mao**

Supporting Information

Prediction of clinical precision chemotherapy by patient-derived three-dimensional bioprinting models of colorectal cancer and its liver metastases

Hang Sun, Lejia Sun, Xindi Ke, Lijuan Liu, Changcan Li, Bao Jin, Peipei Wang, Zhuoran Jiang, Hong Zhao, Zhiying Yang, Yongliang Sun, Jianmei Liu, Yan Wang, Minghao Sun, Mingchang Pang, Yinhan Wang, Bin Wu, Haitao Zhao, Xinting Sang, Baocai Xing, Pengyu Huang*, Huayu Yang*, Yilei Mao**

Figure S1, Supporting Information

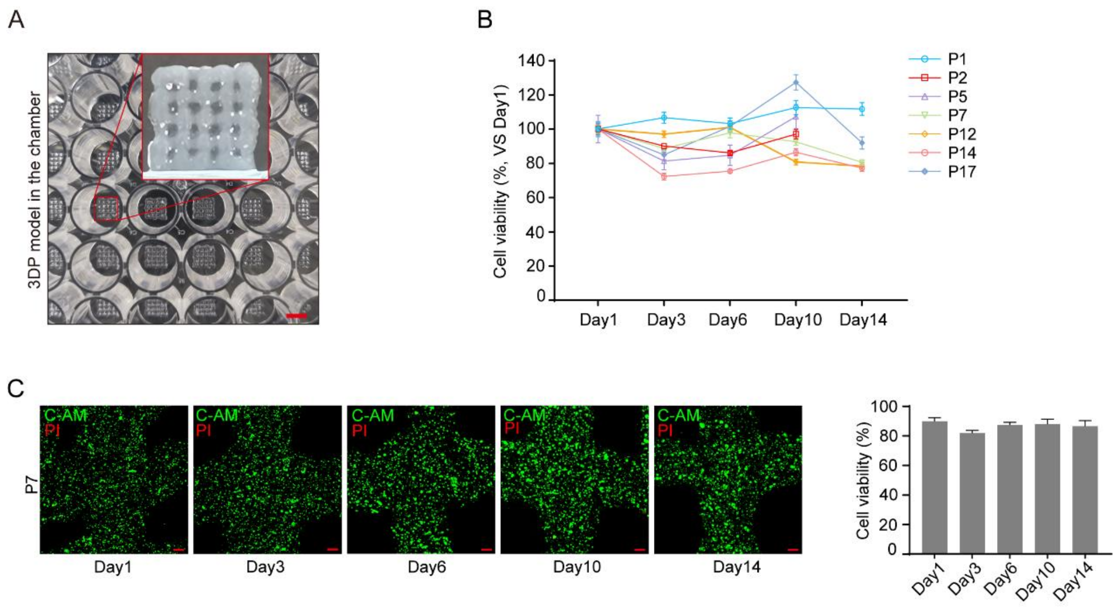

Figure legends

(A) The general appearance of the printed 3DP models.

(B) Cell viability of CRC 3DP models was detected by CellTiter-Glo® 3D Cell Viability Assay on day 1, day 3, day 6, day 10, and day 14 after printing. Data were presented as mean ± SD.

(C) The viability test of primary tumor cells in CRC 3DP models on day 1, day 3, day 6, day 10, and day 14 after printing by calcein-AM and propidium iodide staining. Living cells were labeled in green, and dead cells were observed in red. A Scale bar = 100µm.

Figure S2, Supporting Information

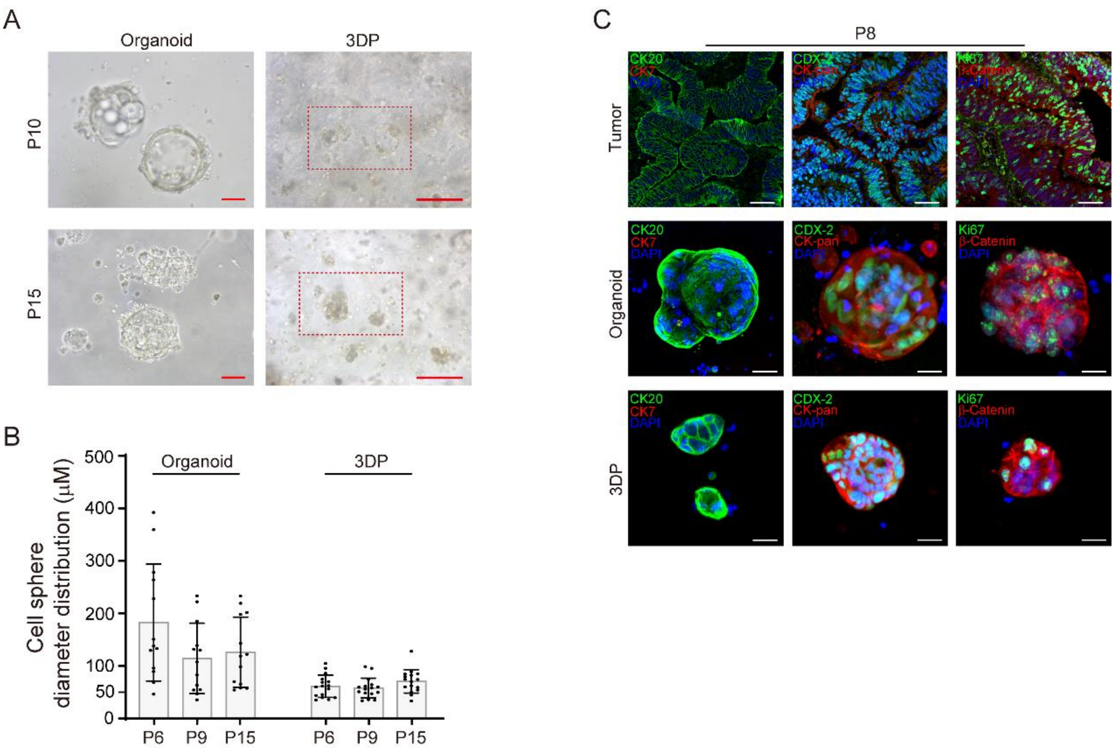

Figure legends

(A) Bright-field images showed CRC 3DP models and PDTOs on day 6 post-bioprinting. Scale bar = 100 μm.

(B) Comparison of cell sphere sizes between CRC 3DP models and PDTOs.

(C) CRC 3DP models and corresponding organoids and parent tumors were co-stained with CK20 (green), CK7 (red), CDX2 (green), CK-pan (red), β-catenin (red), Ki-67(green) to examine the profile CRC biomarkers. DAPI was used to visualize nuclei (blue). Scale bar of tumor, 50 μm. Organoid and 3DP scale bars, 20 μm.

Figure S3, Supporting Information

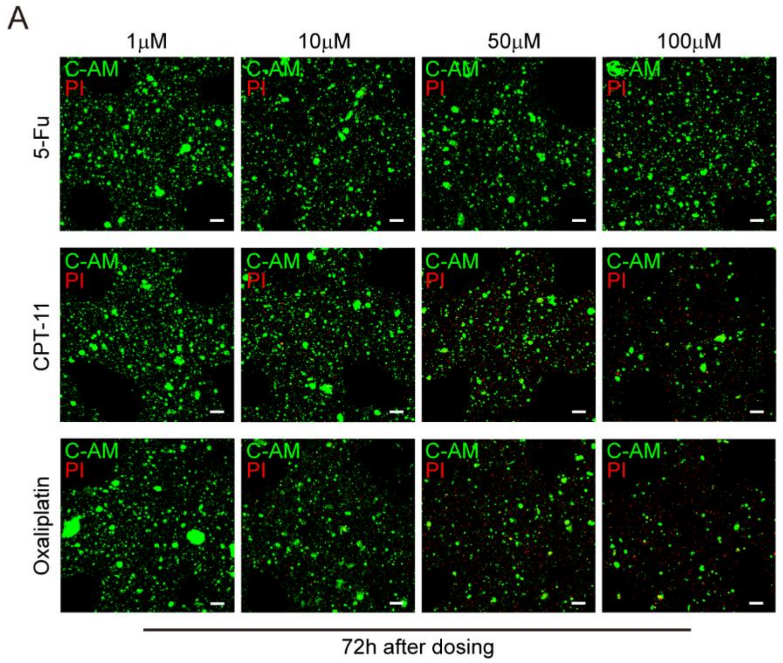

Figure legends

(A) Cell viability staining of CRC 3DP models treated with 5-FU, CPT-11, and oxaliplatin at concentrations of 100 μM, 50 μM, 10 μM, 1 μM, and 0 μM for 72 hours using calcein-AM and propidium iodide staining derived from CRC Patient 12 (P12). Viable cells are indicated in green, while deceased cells are marked in red. Scale bar = 100 μM.

Figure S4, Supporting Information

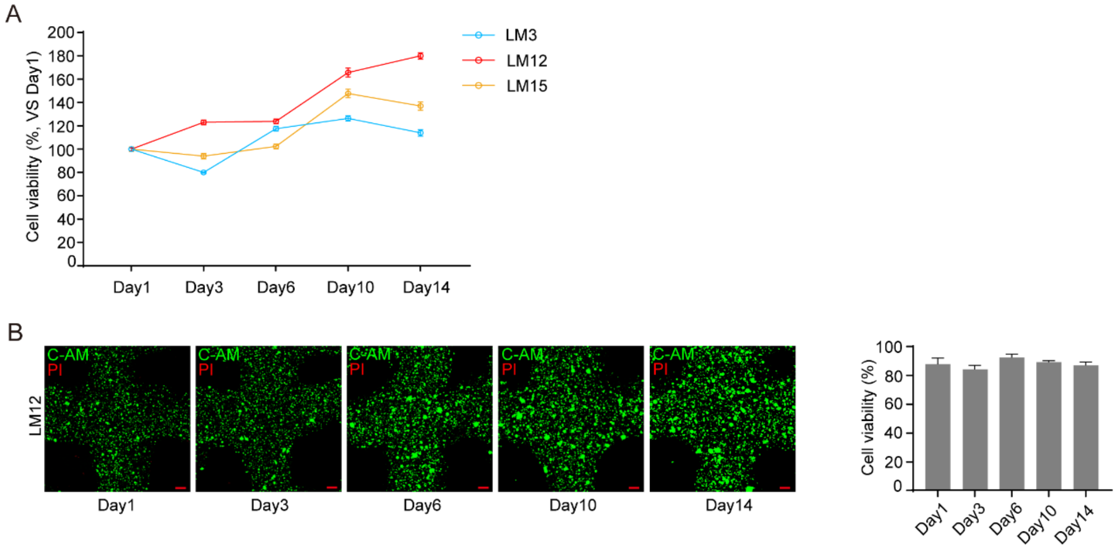

Figure legends

(A) Cell viability of CRLM 3DP models was detected by CellTiter-Glo® 3D Cell Viability Assay on day 1, day 3, day 6, day 10, and day 14 after printing. Data were demonstrated as mean ± SD.

(B) The viability test of primary tumor cells in CRLM 3DP models on day 1, day 3, day 6, day 10, and day 14 after printing by calcein-AM and propidium iodide staining. Living cells were marked as green, and dead cells were observed as red. A Scale bar = 100μm.

Figure S5, Supporting Information

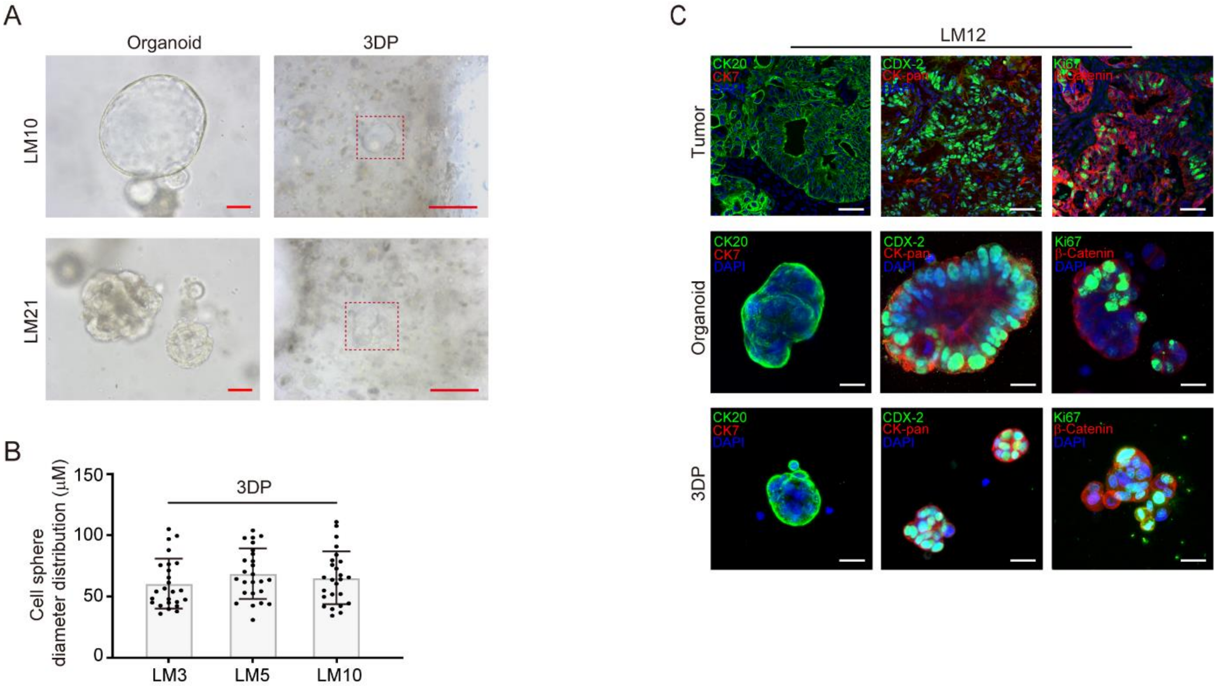

Figure legends

(A) Bright-field images images of CRLM 3DP models and PDTOs on day 6. Scale bar = 100 μm.

(B) Cell sphere diameter distribution in CRLM 3DP models measured under bright-field images.

(C) CRLM 3DP models and corresponding organoids and parent tumors were co-stained with CK20 (green), CK7 (red), CDX2 (green), CK-pan (red), β-catenin (red), Ki-67(green) to examine the profile of CRLM biomarkers. DAPI was used to visualize nuclei (blue). Scale bar of tumor, 50 μm. Organoid and 3DP scale bars, 20 μm.

Figure S6, Supporting Information

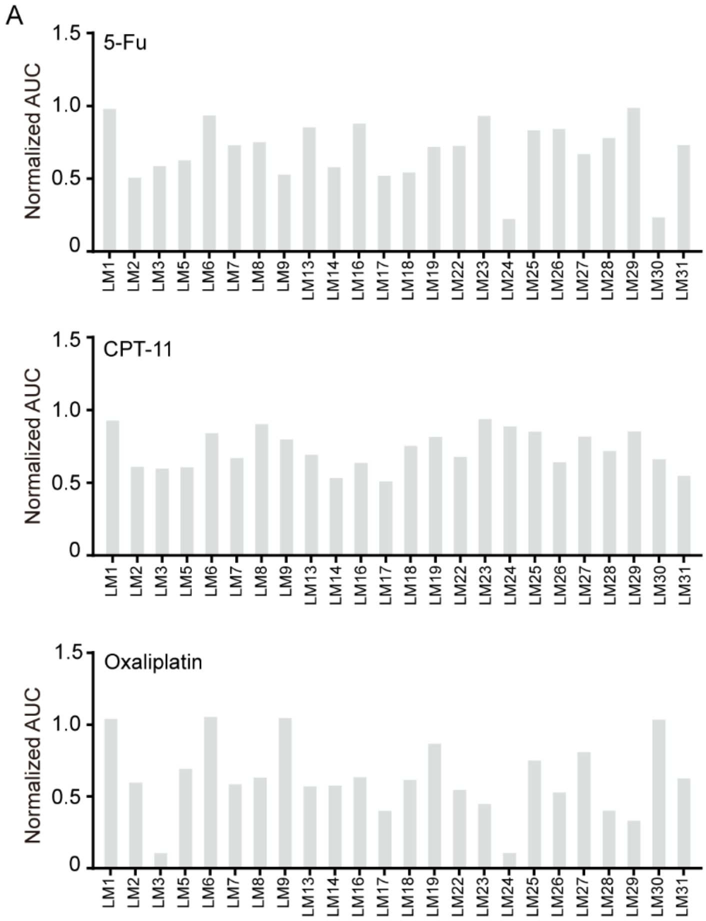

Figure legends

The sensitivity of 24 CRLM 3D-printed models to the chemotherapeutic drugs 5-FU, CPT-11, and oxaliplatin was assessed by determining the normalized area under the curve (AUC) from the corresponding dose-response curves.

Figure S7, Supporting Information

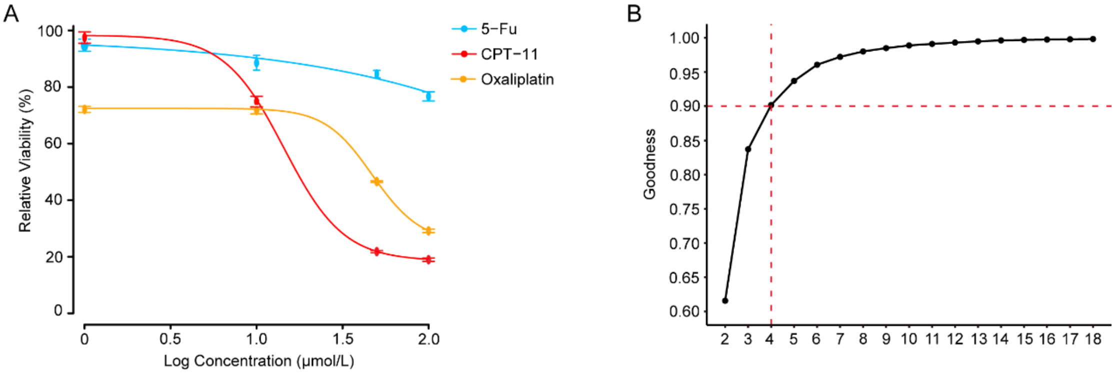

Figure legends

(A) Dose-response curves for drug testing on CRLM 3DP models derived from LM16. Drugs were selected based on clinical guidance for the treatment of colorectal cancer and its liver metastases. A concentration gradient from 0 μM to 100 μM was set. (B) Goodness-of-fit curve to identify the optimal number of breaks for Jenks classification to avoid over-fitting. The classification that generated the minimum number of breaks was used to obtain a goodness-of-fit greater than 90% was adopted (dotted lines).

Supplementary Table 1: Clinical characteristics of patients with colorectal cancer.

	Sex	Age (years)	Histopathology	Primary site	Liver metastasis	Neoadjuvant treatment	Clinical response
P1	female	56	adenocarcinoma	rectum	No	No	-
P2	male	47	adenocarcinoma	rectum	No	No	-
P3	male	82	adenocarcinoma	colon	No	No	-
P4	female	75	adenocarcinoma	rectum	No	No	-
P5	male	84	adenocarcinoma	colon	No	No	-
P6	female	76	adenocarcinoma	rectum	No	No	-
P7	male	72	adenocarcinoma	rectum	No	No	-
P8	female	71	adenocarcinoma	rectum	No	No	-
P9	male	77	adenocarcinoma	rectum	No	No	-
P10	male	80	adenocarcinoma	colon	No	No	-
P11	female	48	adenocarcinoma	colon	Yes	FOLFOX	TRG 3
P12	male	72	adenocarcinoma	colon	No	No	-
P13	male	69	adenocarcinoma	colon	Yes	XELOX	TRG 5
P14	male	55	adenocarcinoma	colon	No	No	-
P15	male	55	adenocarcinoma	colon	No	No	-
P16	female	64	adenocarcinoma	rectum	Yes	XELOX	TRG 3
P17	female	28	adenocarcinoma	rectum	Yes	FOLFOX	TRG 4
P18	female	48	adenocarcinoma	rectum	No	No	-
P19	female	80	adenocarcinoma	colon	Yes	No	-
P20	male	64	adenocarcinoma	rectum	No	No	-
P21	male	44	adenocarcinoma	colon	Yes	FOLFOX	TRG 5
P22	female	52	adenocarcinoma	colon	Yes	FOLFOX	TRG 5
P23	female	64	adenocarcinoma	rectum	No	No	-
P24	male	69	adenocarcinoma	colon	Yes	XELOX	TRG 5
P25	female	70	adenocarcinoma	rectum	No	No	-
P26	female	75	adenocarcinoma	colon	No	No	-
P27	female	59	adenocarcinoma	rectum	No	No	-
P28	male	52	adenocarcinoma	colon	Yes	No	-

P29	female	51	adenocarcinoma	colon	No	No	-
P30	male	63	adenocarcinoma	rectum	No	No	-
P31	female	83	adenocarcinoma	colon	No	No	-
P32	female	74	adenocarcinoma	rectum	No	No	-
P33	male	58	adenocarcinoma	colon	Yes	FOLFIRI	TRG 4
P34	female	55	adenocarcinoma	colon	No	XELOX	TRG 5
P35	male	71	adenocarcinoma	colon	No	No	-
P36	male	71	adenocarcinoma	colon	No	No	-
P37	female	64	adenocarcinoma	rectum	No	No	-
P38	male	55	adenocarcinoma	colon	No	No	-
P39	male	49	adenocarcinoma	colon	No	No	-
P40	male	58	adenocarcinoma	colon	Yes	FOLFOX	TRG 3

Supplementary Table 2: Clinical characteristics of patients with liver metastases.

	**Sex**	**Age (years)**	**Histopathology**	**Primary site**	**Synchronous liver metastasis**	**Neoadjuvant treatment**	**Clinical response**
LM1	female	59	adenocarcinoma	colon	No	FOLFOXIRI	PD
LM2	male	65	adenocarcinoma	colon	No	FOLFOXIRI	PR
LM3	male	71	adenocarcinoma	rectum	No	-	-
LM4	male	45	adenocarcinoma	colon	No	-	-
LM5	female	28	adenocarcinoma	rectum	Yes	FOLFOX	SD/TRG3
LM6	female	80	adenocarcinoma	colon	Yes	-	-
LM7	male	74	adenocarcinoma	colon	No	FOLFOX	SD
LM8	male	44	adenocarcinoma	colon	Yes	FOLFOX	SD/TRG3
LM9	male	56	adenocarcinoma	rectum	No	-	-
LM10	male	54	adenocarcinoma	rectum	No	FOLFOX	PR
LM11	male	64	adenocarcinoma	rectum	No	-	-
LM12	male	53	adenocarcinoma	colon	No	-	-
LM13	male	64	adenocarcinoma	colon	No	XELOX	SD
LM14	female	52	adenocarcinoma	colon	Yes	FOLFOX	SD/TRG3
LM15	female	50	adenocarcinoma	rectum	No	-	-
LM16	male	69	adenocarcinoma	colon	Yes	XELOX	SD/TRG4
LM17	female	67	adenocarcinoma	colon	No	FOLFOX	PR
LM18	male	58	adenocarcinoma	rectum	Yes	FOLFIRI	SD/TRG2
LM19	male	58	adenocarcinoma	colon	No	FOLFOX	PD
LM20	female	45	adenocarcinoma	colon	No	-	-
LM21	male	37	adenocarcinoma	rectum	No	-	-
LM22	male	54	adenocarcinoma	colon	No	-	-
LM23	male	58	adenocarcinoma	colon	Yes	FOLFOX	SD/TRG3
LM24	male	72	adenocarcinoma	colon	No	XELOX	PR
LM25	male	51	adenocarcinoma	colon	No	XELOX	PD
LM26	female	69	adenocarcinoma	colon	No	FOLFIRI	SD
LM27	female	71	adenocarcinoma	rectum	No	XELOX	SD

LM28	male	60	adenocarcinoma	colon	No	FOLFOX	SD
LM29	male	77	adenocarcinoma	colon	No	5-FU	PD
LM30	female	63	adenocarcinoma	colon	No	FOLFOX	SD
LM31	male	76	adenocarcinoma	colon	No	FOLFOX	SD
